# Broad ligament ectopic pregnancy at 18 weeks: diagnosis and management in a resource-limited setting (a case report)

**DOI:** 10.11604/pamj.2023.46.40.39598

**Published:** 2023-09-28

**Authors:** Madye Ange Ngo Dingom, Jovanny Fouogue Tsuala, Junie Annick Metogo Ntsama, Wilfried Loïc Meukem Tatsipie, Diane Estelle Kamdem, Jean Marie Alima, Félix Essiben

**Affiliations:** 1 Obstetrics and Gynaecology Department, Central Hospital of Yaoundé, Yaoundé, Cameroon,; 2 Faculty of Medicine and Pharmaceutical Sciences, University of Dschang, Dschang, Cameroon,; 3 Faculty of Medicine and Biomedical Sciences, University of Yaoundé I, Yaoundé, Cameroon

**Keywords:** Broad ligament, ectopic pregnancy, diagnosis, case report

## Abstract

Broad ligament ectopic pregnancy is a relatively rare condition described in the literature. We did not find enough data concerning this subject in Cameroon. It is therefore important to know about its existence because late management can lead to increased maternal mortality. This paper reports the case of a 22-year-old lady at 18 weeks gestation who had generalized abdominal pain for two months. Prior to her arrival at our service, an abdominal and pelvic ultrasound done revealed a viable singleton 18-weeks intra-abdominal pregnancy with a moderate amount of abdominal fluid collection. The diagnosis of haemorrhagic shock complicating an abdominal pregnancy at 18 weeks of gestation was retained. An emergency laparotomy was done, and a right total salpingectomy and oophorectomy with resection of the right broad ligament were carried out. After surgery, dissection of the mass revealed a non-viable male foetus weighing 218 grams. In conclusion, there´s a very high morbidity and mortality rate associated with broad ligament pregnancies. Due to the fact that there is late access to antenatal care, the prognosis of pathologic pregnancies is endangered.

## Introduction

Abdominal pregnancy is a rare type of ectopic pregnancy, with an incidence varying between 1: 10,000 to 1: 30,000 pregnancies [1]. An ectopic pregnancy occurs in 2% of all pregnancies, with 95% located in the fallopian tube [2]. Abdominal pregnancies could be life-threatening with maternal mortality eight times greater than the usual tubal ectopic pregnancies [3]. Broad ligament pregnancy is a very rare variation of ectopic pregnancies and belongs to the group of abdominal pregnancies. Clinical and Radiologic (ultrasound) diagnosis is not easy. This case is unique because it´s most certainly the first described in our community, hence management protocols have not yet been established.

## Patient and observation

**Patient information:** a 22-year-old housewife, G3P2002, at 18 weeks gestation according to an obstetric ultrasound performed on the day of admission. She was admitted at the emergency unit for generalised abdominal pain of gradual onset about 2 months prior to consultation. The pain was initially at the right iliac fossa, then progressively became generalised. The pain was piercing in character and constant, associated with abdominal distension, several episodes of late postprandial vomiting and gradually worsening asthenia. Aggravation of the pain prompted the excessive use of non-steroidal anti-inflammatory drugs with minimal relief of pain. Concerning the past history of the patient, there was no notion of past intra-uterine manipulations, her medical and surgical history were unremarkable.

**Clinical findings:** on physical examination, we had an ill-looking patient, conscious, well oriented in time and space. Vital signs: blood pressure was undetectable, threading pulses at 144 beats per minute, respiratory rate was at 36 breaths per minute, the temperature at 36.8 degree celsius. The conjunctivae, skin and mucous membranes were pale, with cold extremities and a capillary refill time greater than 30 seconds. On heart exam, the heart sounds were regular and tachycardic, with no murmurs. Examination of lungs, there was polypnea, without crackles. The abdomen was distended and mobile with respiration. There was generalised tenderness of the abdomen on palpation, bowel sounds were absent. Speculum examination showed a macroscopically normal cervix with a bluish coloration and there was no discharge from the endocervix. The vaginal walls were macroscopically normal. On digital vaginal examination, the cervix was long posterior and closed, the uterus was difficult to appreciate because of the pain; the Douglas pouch was bulging and very tender; fingers were clean. Digital rectal examination revealed tenderness in the posterior pouch of Douglas. Paracentesis done brought 2 ml of bright red nonclotting blood. The rest of the clinical examination was unremarkable.

**Timeline:** the patient had an onset of right iliac fossa pains two months prior to admission, that gradually became generalised. This was associated with abdominal distension, vomiting and asthenia, leading to self-medication. This provided only mild relief, prompting consultation at the emergency unit of the main maternity of the Central Hospital Yaoundé.

**Diagnostic assessment:** an abdominal and pelvic ultrasound performed prior to her arrival in our unit revealed she had a viable 18 weeks single intra-abdominal pregnancy with a moderate amount of intra-abdominal fluid collection. An emergency full blood count showed a haemoglobin level of 6 g/dl, her blood group was O rhesus positive. Diagnosis: the notion of amenorrhoea, pelvic pain, and hemoperitoneum at 18 weeks sonographic gestational age, made us retain the diagnosis of ruptured abdominal pregnancy complicated by haemorrhagic shock. As differential diagnosis, we thought of a ruptured cornual pregnancy of 18 weeks complicated by haemorrhagic shock. Prognostic characteristics when applicable: the prognosis depended on the patient´s age, her initial hemodynamic state, and the delay in management.

**Figure 1:**
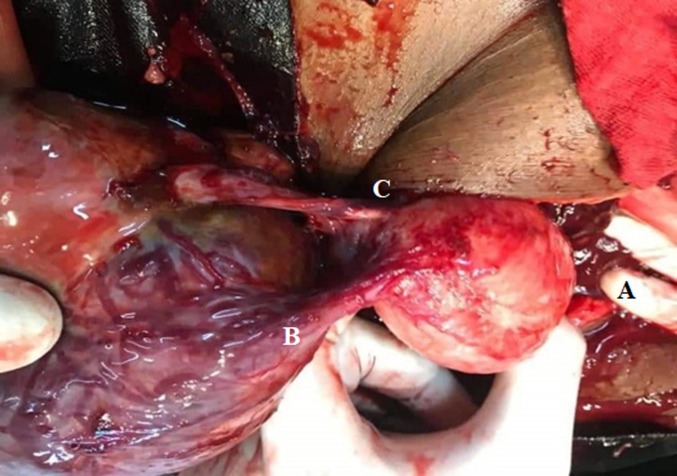
pre-operative findings: uterus (A), right lateral mass (B), and right ovary (C)

**Figure 2:**
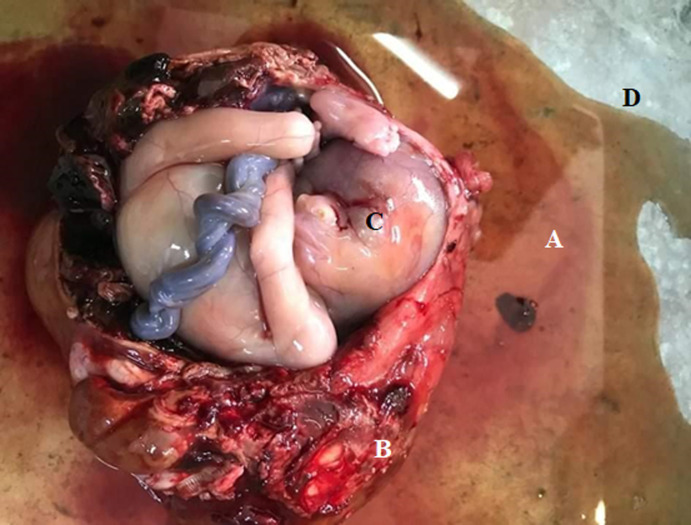
macroscopic post-operative findings after opening the mass; fetus (A); placenta (B); umbilical cord (C); amniotic fluid (D)

**Therapeutic intervention:** we initially started with resuscitation measures. We placed two large bore intravenous lines with 1000 ml of 0.9% normal saline, and an exploratory laparotomy was indicated and done. We made a paramedian incision of 15 cm. After opening of the skin and subcutaneous tissues, we gradually crossed the rectus abdominis muscles and reached the parietal peritoneum. Opening of the parietal peritoneum revealed a right lateral-uterine fissured mass of the right broad ligament measuring 120 x 140 mm. The right fallopian tube and ovary were not visualised, both were attached to the mass. Only the right tubal fimbriae were visible, as shown in Figure 1. The hemoperitoneum was 1500 ml. The contra-lateral adnexa and uterus were macroscopically normal. We performed a right total salpingectomy and oophorectomy with resection of the right broad ligament. The patient received 2 units of packed red blood cells during the surgery. After the surgery, dissection of the mass revealed the presence of male death foetus of weight 218 grams as shown in Figure 2. Histopathological analysis was requested but not done by the patient.

**Follow-up and outcomes:** after surgery, the patient was admitted to the recovery room where she spent two days before being transferred to the hospitalisation ward. She received one unit of packed red blood cells post-operatively. She was put on intravenous antibiotics (ceftriaxone 1 g every 12 hours), fluids (2500 ml every 24 hours) and analgesics (paracetamol 1000 mg every 6 hours). During postoperative monitoring, the pulse and respiratory rate normalized within twelve hours. Oral relay was initiated on day 2 post-operative, consisting of antibiotherapy; combination of ofloxacine/tinidazole 1 tablet every 12 hours for 10 days, and paracetamol 1000 mg 1 tablet every 8 hours for 3 to 5 days. Day 8 postoperative was marked by favourable evolution hence patient was discharged. The postoperative appointment given 15 days after discharge was unremarkable.

**Patient perspective:** clinical progress was favourable and patient´s symptoms improved. No complications were reported following the surgery. Furthermore, the patient expressed her gratitude for prompt management and thanked the medical staff for their competence.

**Informed consent:** we obtained consent from the patient for the write-up and publication of this case report.

## Discussion

Intra-ligamentous pregnancy is a relatively rare entity in the group of abdominal ectopic pregnancies. Its incidence is estimated to be 1 in 300 extra uterine pregnancies. Abdominal pregnancy can occur in any part of the abdomen; most commonly in the pouch of Douglas but rarely in the broad ligament [4]. Maternal and foetal mortality is significant. In the main maternity unit of the Yaounde Central Hospital, we have recorded up to 400 cases of extra uterine pregnancies, over the last 10 years but have only recorded one case of intra-ligamentous pregnancy i.e. an incidence of 1/400.

It usually results from trophoblastic invasion of the tubal pregnancy through the tubal serosa and into the mesosalpinx, with secondary implantation between the broad ligament leaflets [5]. In this case, it is a secondary abdominal pregnancy. It may also result from primary implantation in the broad ligament. The first theory could explain the occurrence of the intra-ligamentous pregnancy in our case, due to the fact that on macroscopic examination only the right fimbriae were visualised. The abdominal pain observed early in these pregnancies is thought to be due to separation of the placenta and due to minimal hemoperitoneum [5]. This was observed in our patient who had described a first episode of abdominal pain two months earlier, with mild relief on non-steroidal anti-inflammatory drugs. Early occurrence of complications such as moderate to severe abdominal pain or vaginal spotting allows the diagnosis to be made early. The resulting intra-abdominal haemorrhage may be revealed by an acute abdomen or a state of shock. These complications are associated with a maternal and perinatal mortality of 40% and 95% respectively [6]. In our case, the patient presented in a state of shock and the obstetric ultrasound done before arrival at our unit revealed the foetus was alive.

The diagnosis was made during ultrasound and confirmed after surgery. Abdominal ultrasound is a reliable diagnostic tool in some studies [7]. Magnetic resonance imaging (MRI) provides more information to ultrasound in the diagnosis of intra-ligamentous pregnancy [7]; however, its high cost in developing countries causes medical practitioners to limit themselves to clinical assessment, which is highly presumptive, and to abdominal ultrasound. Early diagnosis and immediate operative management are necessary to avoid poor prognosis.

In our case, the life-threatening factor was the acute abdomen and the haemorrhagic shock. A right total salpingectomy and oophorectomy were performed. This procedure was also performed in the two patients described by Sharma *et al*. [8], all of whom were hemodynamically unstable. However, it is recommended that the placenta be left in situ if removal could be life-threatening. The administration of methotrexate combined with plasma β-HCG, monitoring and ultrasound can ensure follow-up. However, in stable patients and in early pregnancy, laparoscopy is preferable [9]. Embolization of the lateral sacral artery has been described in the management of intra-ligamentous pregnancies.

Difficulties in accessing health care delay optimal patient management. Our patient was no exception. She decided to take anti-inflammatory drugs at the onset of pain due to a lack of finances. Regular quality antenatal consultations could help avoid maternal and foetal complications, as the mortality rate remains significant. In literature, only a few cases of live foetuses have been described [10]. Early antenatal consultation helps in the diagnosis of pregnancy and the identification of its location. In an emergency situation, the best possible management plan must be implemented.

## Conclusion

The occurrence of a pregnancy in the broad ligament is rare in our specialty. Its diagnosis is clinical and paraclinical, especially if the ultrasound is done early and by an experienced radiologist. Its management is similar to that of abdominal pregnancy. The maternal prognosis can be poor when the management is not optimal in terms of surgery, anaesthesia, resuscitation, and in the absence of blood products. Hence the importance of first-trimester ultrasound during antenatal contacts and if such a case is observed, it is necessary to make sure the patient is managed in a structure with an adequate technical platform (operating theatre, anaesthesia-intensive care, visceral surgeons, and gynaecologists).
